# Cancer Spectrum, Family History of Cancer and Overall Survival in Men with Germline *BRCA1* or *BRCA2* Mutations

**DOI:** 10.3390/jpm11090917

**Published:** 2021-09-15

**Authors:** Florian Reichl, Daniela Muhr, Katharina Rebhan, Gero Kramer, Shahrokh F. Shariat, Christian F. Singer, Yen Y. Tan

**Affiliations:** 1 Department of Obstetrics, Gynecology and Comprehensive Cancer Center, Medical University of Vienna, Waehringer Guertel 18-20, 1090 Vienna, Austria; florian.reichl@meduniwien.ac.at (F.R.); daniela.muhr@meduniwien.ac.at (D.M.); christian.singer@meduniwien.ac.at (C.F.S.); 2 Department of Urology, Medical University of Vienna, Waeringer Guertel 18-20, 1090 Vienna, Austria; katharina.rebhan@meduniwien.ac.at (K.R.); gero.kramer@meduniwien.ac.at (G.K.); shahrokh.shariat@meduniwien.ac.at (S.F.S.); 3 Institute for Urology and Reproductive Health, Sechenov University, 119991 Moscow, Russia; 4 Department of Urology, Weill Cornell Medical College, New York, NY 10065, USA; 5 Department of Urology, University of Texas Southwestern, Dallas, TX 75390, USA; 6 Department of Urology, Second Faculty of Medicine, Charles University, 15006 Prague, Czech Republic

**Keywords:** hereditary cancer, men, BRCA mutations, cancer spectrum, parent of origin, family history

## Abstract

BACKGROUND: Men with germline *BRCA1/2* mutations are not well studied compared to their female counterparts. This study evaluates the cancer characteristics, family history of cancer, and outcomes of male *BRCA1/2* mutation carriers. METHODS: All men with germline *BRCA1*/*2* mutations who attended genetic assessment between October 1995 and October 2019 at the Medical University of Vienna were identified. Clinicohistopathological features, family history of cancer, and outcomes were assessed by mutation status. RESULTS: Of the 323 men included, 45 (13.9%) had a primary cancer diagnosis, many of whom were *BRCA2* carriers (75.5%). Breast cancer (BC) was the most common cancer (57.8%) followed by prostate cancer (15.6%). Invasive ductal carcinoma and hormone receptor positive tumors were the most common. Among 26 BC-affected patients, 42% did not have any relatives with cancer. Parent of origin was only known in half of the 26 men, with 42% of them inherited through the maternal lineage versus 8% through the paternal. *BRCA2* carriers and those with a family history of BC had worse overall survival (20 y vs. 23 y *BRCA1* carriers; P = 0.007; 19 y vs. 21 y for those without family history of BC; P = 0.036). CONCLUSION: Male BRCA2 carriers were most likely to develop cancer and had worse prognosis. In our dataset, BC was the most common cancer, likely due to referral bias. Not all mutation carriers present with BC or have a family history of cancer to warrant genetic testing.

## 1. Introduction

*BRCA1* and *BRCA2* genes are inherited in an autosomal dominant fashion. Both men and women have an equal chance of inheriting either of these genes and could pass them on to their daughters or sons. While there are numerous studies investigating the cancer risks and outcomes of female carriers [1,2,3], the cancer characteristics of male mutation carriers are based on just a handful of studies [1,2,4,5,6]. It has been estimated that the average man has less than 1% lifetime risk of developing breast cancer (BC) [7], but for men who harbor a *BRCA1-* or *BRCA2-* mutation, this risk amplifies significantly [8]. The age adjusted cumulative risk of BC in men with *BRCA1-*mutation at age 70 years was 1.2% (95% CI = 0.22% to 2.8%) and that for *BRCA2-*mutations was 6.8% (95% CI = 3.2% to 12%). Male BC, although rare, has been increasing over the past few decades [9,10]. There is, in parallel, an increasing appreciation of differences in the tumor biology and clinical behavior between female and male BC, thereby highlighting the need for studies focused on this unique population.

Besides BC, male germline mutation carriers also have an increased lifetime risk for prostate cancer with a cumulative lifetime risk of 29% (95% CI = 17% to 45%) for *BRCA1*-mutation carriers and 60% (95% CI = 43–78%) for *BRCA2*-mutation carriers [11] compared to a lifetime risk of 16% of the general population [12,13]. *BRCA2-*mutation carriers also have an up to 5% increased risk for pancreatic cancer [5,14]. Recent studies reported BC and prostate cancer to be the most commonly diagnosed cancers among male *BRCA* carriers [4,5,15,16]. Other frequently reported cancers observed with a *BRCA* mutation are colorectal, pancreatic, bladder, stomach, and melanoma [4,5,17]. Several studies on the survival and mortality of *BRCA1*- and *BRCA2*-mutation carriers have come to a similar conclusion, associating a poorer overall survival and an increased mortality with a *BRCA2*-mutation [15,18,19]. A recent study, focusing on men only, also reported that *BRCA2* mutations were associated with family history of breast/ovarian cancer [20]. Despite these findings, men were less likely to be identified or referred for genetic testing [21]. When patients who inherited the mutation paternally were diagnosed with cancer, they were also less proactive in managing their risk than those who inherited the mutations maternally [22]. 

Men with germline *BRCA1/2* mutations are not well studied compared to their female counterparts. The lack of data has led to poor evidence to drive recommendations regarding early cancer detection and risk reduction in this population. This study aimed to elucidate the cancer spectrum, family history of cancer, and outcomes of male patients with *BRCA1/2*-mutations. 

## 2. Materials and Methods

### 2.1. Patients

High-risk healthy or cancer-affected men who attended genetic counselling and testing at the Department of Obstetrics and Gynaecology at the Medical University of Vienna between October 1995 and October 2019 were identified for this study.

Patients are eligible to undergo genetic testing if they fulfil one of the following family history criteria: (1) three BC cases, (2) two BC cases before age 50, (3) one BC case before age 35, (4) one BC case and one ovarian cancer at any age, (5) two ovarian cancer cases at any age, (6) one bilateral BC case in first-degree relative diagnosed before age 50, (7) male BC, or the following personal medical history: (8) triple negative breast cancer diagnosed before age 60 or when therapeutically indicated, or (9) epithelial ovarian cancer (including fallopian tube and peritoneal carcinoma). Following eligibility, the patients were asked for informed consent and to provide a blood sample for genetic testing. All patient data, such as year of birth, primary/secondary/tertiary tumor sites, date of genetic testing, age at cancer diagnoses, age/date at last follow-up, family history of cancer, health outcomes, and histopathology reports were collected.

### 2.2. Genetic Testing 

Genetic testing for *BRCA* mutation has been conducted at the Medical University of Vienna/Vienna General Hospital since 1995. Genomic DNA was isolated from patients’ blood specimens using standardized methods and quantified by Qubit fluorimeter (Life Technologies, Carlsbad, CA, USA) and/or a NanoDrop spectrophotometer (Thermo Fisher Scientific Inc., Waltham, MA, USA). Targeted mutation analysis was first performed with denaturing high-performance liquid chromatography (dHPLC) and was replaced by Sanger sequencing in 2007. Multiplex ligation-dependent probe amplification (MLPA) was performed subsequently to identify large detections and duplications, and to further confirm mutations in the suspected gene. Next generation sequencing (NGS) replaced Sanger sequencing in 2015, and multigene panel testing was performed using the Illumina TruSight Cancer panel on the MiniSeq instrument according to the manufacturer’s instructions (Illumina, San Diego, CA, USA). We shortlisted 18 cancer genes for testing, i.e., *BRCA1*, *BRCA2*, *ATM*, *CHEK2*, *PALB2*, *RAD51C*, *RAD51D*, *BRIP1*, *NBN*, *MLH1*, *MSH2*, *MSH6*, *PMS2*, *EPCAM*, *TP53*, *PTEN*, *STK11*, and *CDH1*. Data analysis was performed using Sophia DDM^®^ software (Sophia Genetics). Once a mutation was identified, it was further classified, based on the probability of pathogenicity, for further risk assessment using Clinvar (https://www.ncbi.nlm.nih.gov/clinvar/). The variants are classified into one of the five categories, ranging from class 1 (not pathogenic) to class 5 (pathogenic variants, PV) [23]. Only class 4 (likely pathogenic) and class 5 (pathogenic) variants were included in this study.

### 2.3. Statistical Analysis

Descriptive statistics such as percentages, mean, median, range, and 95% confidence interval (CI) were reported for the entire study cohort. The primary objective was to compare the distribution of cancer and to report tumor characteristics, family history of cancer between *BRCA1* and *BRCA2* carriers. Seven double mutations, i.e., *BRCA1* and *BRCA2* and were excluded from the final analyses. Comparisons between cancer types, categorical age, parent of origin, family history of cancer, and *BRCA* mutation status were conducted using Chi-square or Fisher’s test (expected cell frequency < 5). The median age at diagnosis was compared between mutation status using the Mann–Whitney test. Follow-up time was calculated using date of death or last date of follow-up and date of diagnosis (if patient was affected by cancer) or date of genetic testing. Overall survival estimates were calculated using the Kaplan–Meier analysis; difference between groups were compared with log-rank test. All reported P-values were two-sided, and a value of <0.05 was considered statistically significant. All statistical analyses were conducted using SPSS, version 26.0.0 (SPSS Inc., Chicago, IL, USA).

## 3. Results

**Figure 1:**
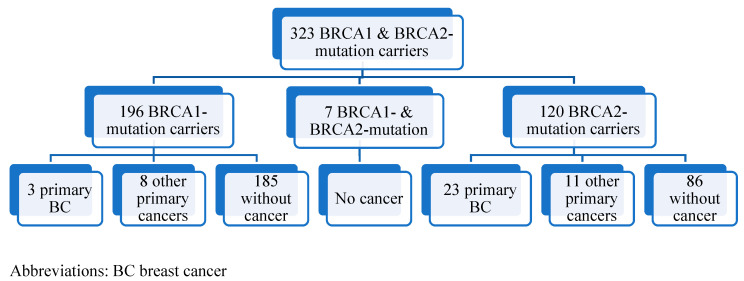
Cohort selection and inclusion.

**Figure 2:**
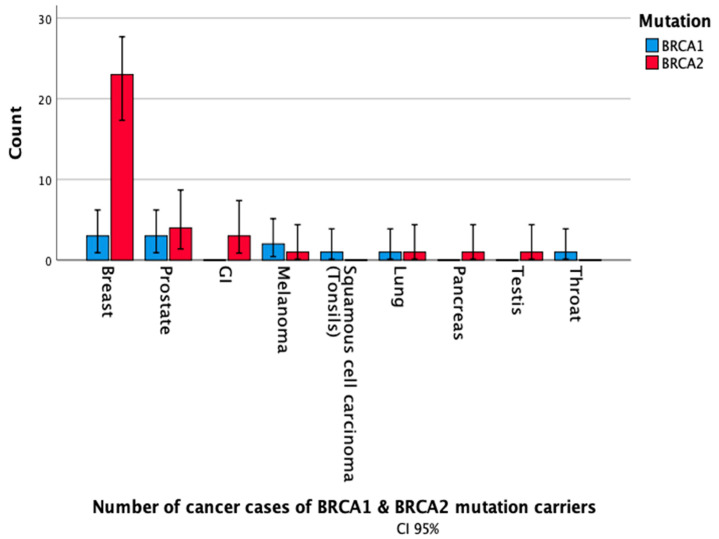
Primary cancer cases by mutation status.

This study included 323 men with confirmed germline *BRCA1* or *BRCA2* pathogenic (class 5) or likely pathogenic (class 4) mutations (Table 1). Of the 323 men, 196 (60.6%) carried a *BRCA1*-mutation, 120 (37.2%) carried a *BRCA2*-mutation, and the remaining 7 (2.2%) carried both mutations (Figure 1). Forty-five (13.9%) had at least one cancer diagnosis, the majority of whom harbored a *BRCA2* mutation (75.5%) (Table 2, Figure 2). The median age at diagnosis for any first cancer was 59 years (range 39–88 years). The median duration of follow-up was 4 years after genetic testing and the median age at testing was 42 years (range 28–56 years).

Of all cancers diagnosed, BC was the most common (N = 26, 57.7%; 3 *BRCA1* and 23 *BRCA2*; P < *0*.001), followed by prostate cancer (N = 7, 15.6%; 3 *BRCA1* and 4 *BRCA2*). Other cancers include gastrointestinal, skin, pancreas, throat, lung, and testicular cancer (N = 12, 26.7%). Twelve patients (3.7%) had a second primary cancer, with breast being the most common second cancer in *BRCA2* carriers (41.7% vs. none in *BRCA1* carriers, P = 0.007). Five had a third primary cancer. 

Primary breast tumor characteristics were also assessed and compared between mutation status (Table 3). Not all histopathology data was available for all affected. Invasive ductal carcinoma and hormone receptor positive (ER = 93%, PR = 81%) were the most common subtype observed. All cancers diagnosed were unilateral breast cancers, treated with modified radical mastectomy, and only three patients opted for prophylactic bilateral mastectomy; reports of the other patients regarding prophylactic measures were not available. The majority of the cases also occurred on the left side. Notably, four patients who were diagnosed with first BC and subsequently developed a second BC had a *BRCA2* mutation (Supplementary Table S1). There were no statistically significant differences observed in the primary breast tumor characteristics between mutation groups.

For the overall study cohort, the median age at first BC diagnosis was 58 years (range 39–82 years) and median age at first prostate cancer diagnosis was 56 years (range 50–63 years) (Table 4). *BRCA2* carriers were diagnosed earlier with breast cancer at 57 years compared to *BRCA1* carriers at 62 years. Prostate cancer diagnosis was also earlier in *BRCA2* carriers at 52 years compared to *BRCA1* carriers at 58 years. The differences observed for both cancers were, however, not statistically significant. The age at diagnosis of cancers other than breast and prostate was 65 years in *BRCA2* carriers versus 67 years in *BRCA1* carriers. 

Our results also show that *BRCA2* mutation carriers had a significantly more first- and second-degree relatives with BC and ovarian cancer. However, among 26 men who were affected with first BC, 42% did not have any relatives with cancer (11/26; 2 *BRCA1* and 9 *BRCA2*; P = 0.56). When looking at the parent-of-origin (maternally versus paternally inherited *BRCA* mutation) (Table 5), 11 *BRCA* mutation carriers (42%) were inherited through the maternal lineage versus 2 through the paternal (8%). Although no significant difference was observed between groups, the majority of *BRCA*-associated BC and prostate cancer cases as well as healthy mutation carriers were commonly identified from the maternal lineage.

**Figure 3:**
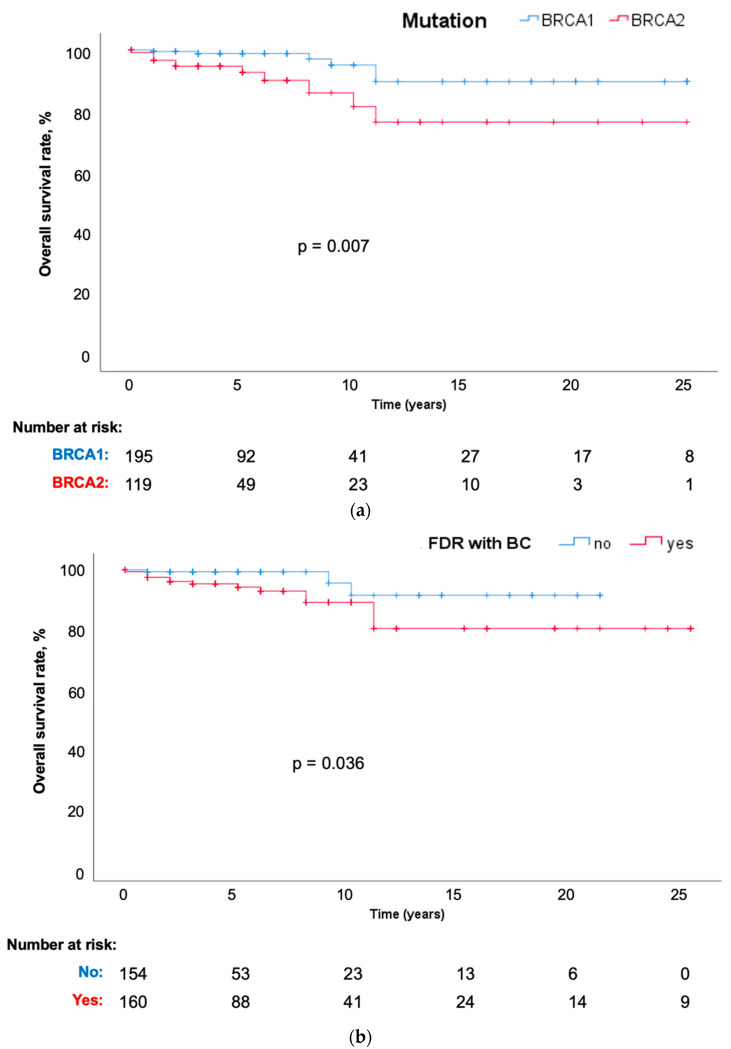

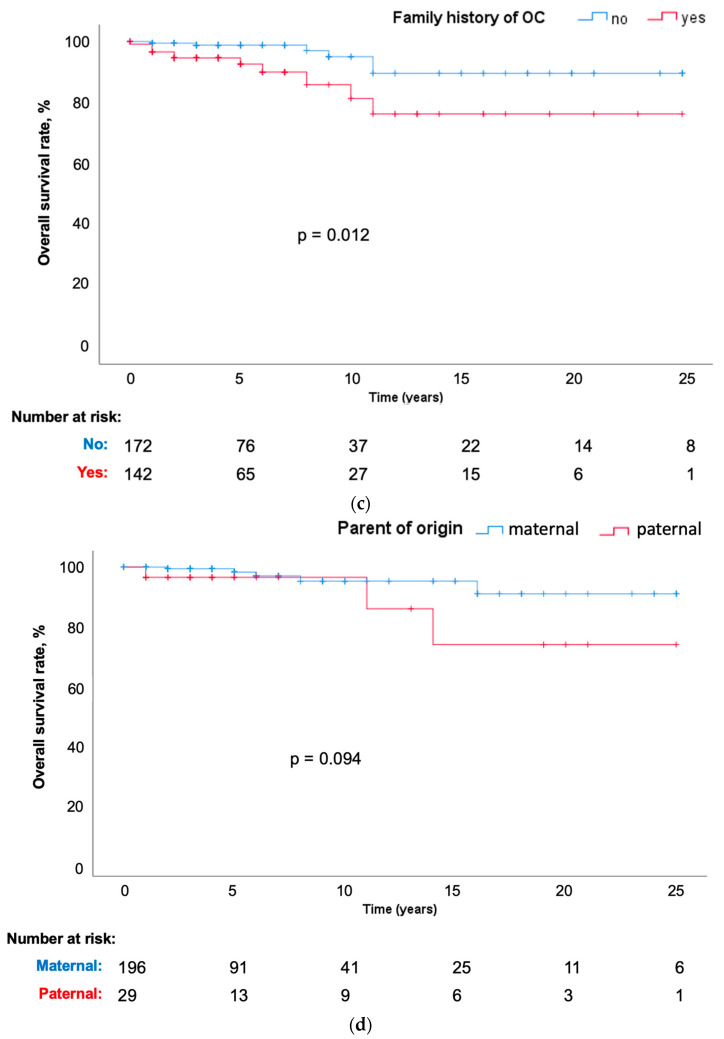
(**a**) OS by mutation type. (**b**) OS and family history of BC. (**c**) OS and family history of ovarian cancer. (**d**) OS and parent of origin.

*BRCA2* mutation carriers were at a survival disadvantage compared to *BRCA1* mutation carriers (20 vs. 23 years, respectively; P = 0.007; Figure 3a). There were 18 deaths overall (6 *BRCA1* and 12 *BRCA2*). Interestingly, about 60% of the deaths observed in *BRCA2*-mutation carriers were related to breast and/or other cancers but only 33% of *BRCA1*-mutation carriers died from breast and/or other cancers. Compared to mutation carriers without a family history of cancer, those with a first-degree relative diagnosed with BC had a significantly poorer overall survival (19 vs. 21 years; P = 0.036; Figure 3b). The same was true for those with a family history of ovarian cancer (21 vs. 23 years; P = 0.012; Figure 3c). There was no significant difference in overall survival observed between those with maternal versus paternal inheritance (21 vs. 23 years, respectively; P = 0.094; Figure 3d).

## 4. Discussion

Men with germline *BRCA* mutations are often underrepresented in clinical studies. To our knowledge, this is the first study investigating male *BRCA1/2* carriers in Austria. The results from our study show that breast and prostate cancer are the most common type of cancers diagnosed, particularly in *BRCA2* carriers, despite the larger proportion of *BRCA1* carriers in our study cohort. Our findings are similar to two other studies investigating the cancer spectrum of male *BRCA1/2*-mutation carriers, where a higher number of overall cancers as well as BC were observed in patients with a *BRCA2*-mutation [5,20]. In our study cohort, no significant difference was observed between age at BC onset and mutation status. However, a later age at diagnosis was observed in male mutation carriers as compared to female mutation carriers (who are usually diagnosed before 50 years; *BRCA1*: 43.6 y vs. *BRCA2*: 45.2 y) [24]. This suggests that men with pathogenic *BRCA* variant experience a later cancer onset as compared to their female counterpart, but the onset is earlier compared with the general male population, which has been reported to be at 64 years [25]. Prostate cancer was also diagnosed earlier in this cohort (*BRCA1*: 58 y vs. *BRCA2*: 52 years) as compared to the general population at 67 years. A similar finding was reported by the IMPACT study where *BRCA2* mutation carriers were associated with a higher incidence of prostate cancer and diagnosed at a younger age as compared to non-carriers [26]. Our study, in line with others [25,27,28], also showed male BC tumors to be mostly estrogen- and progesterone-receptor positive. 

Interestingly, the seven patients with double mutations did not have any cancers diagnosed. This may be due to the later age at onset of breast or prostate cancer but the relatively early age at testing of our cohort (range 18–75 years; 5/7 patients were <50 years). The patients were followed-up biennially like all other participants in the study. However, the reporting of (new) cancers are highly dependent on the patients themselves and, in this instance, it is possible that under-reporting may have occurred, but we were not able to confirm it with other family members. The next follow-up will be performed at the end of the year and perhaps new information will emerge then.

Of the 26 *BRCA2*-cancer affected men, only 3 underwent prophylactic mastectomy. There was no information available for the remaining patients likely due to the retrospective study design where not all data were systematically collected. For women, the cumulative lifetime risk 20 years after breast cancer diagnosis is 40% (95% CI, 35–45%) for *BRCA1* and 26% (95% CI, 20–33%) for *BRCA2* carriers (hazard ratio [HR] for comparing *BRCA2* vs. *BRCA1*, 0.62; 95% CI, 0.47–0.82; P = 0.001 for difference) [3], and is reduced in women who take tamoxifen or who undergo oophorectomy. There is a paucity of data regarding the cumulative lifetime risk or the effectiveness of tamoxifen or risk-reducing bilateral or contralateral mastectomy for affected males. They are generally discouraged due to the low absolute risk of breast cancer [11]. Nevertheless, the effectiveness of prophylactic surgery and chemoprevention for male patients would be an area of interest for future studies.

*BRCA2* mutation carriers had significantly more first- and second-degree relatives with breast and ovarian cancer. Interestingly, not all mutation carriers present with breast or prostate cancer or have a family history of cancer to warrant genetic testing. It is also worth noting that a large proportion of these individuals were identified from the maternal side of the family. Our data suggests that women with multiple affected females in their families are more likely to be referred for genetic testing despite autosomal inheritance. Other reasons could be that men were less receptive to testing or less likely to be tested. Marabelli et al. showed that far fewer men than women are being tested in European countries and that broader genetic data could beneficially contribute to an improved management of disease and increase treatment options [29]. This could be due to physician bias where the *BRCA* genes are perceived to be associated with female breast and ovarian cancer [30] and the lack of awareness that men are equally likely to inherit the *BRCA* gene and to be affected by cancer. Although recent studies found no parent-of-origin effect on breast cancer risk of *BRCA1*/2 mutation carriers [31,32], it has been shown that the recording and interpretation of family history on the paternal side are often underappreciated or neglected [33] and information or the seriousness of the genetic test results are poorly communicated [34]. A recent study further examined a group of *BRCA-*positive women’s coping responses and psychosocial burden based on parent of origin and observed proactive responses among maternally inherited cases compared to reactive responses in paternally inherited cases [22]. These studies and our findings further underline the importance of unbiased pedigree analysis to determine cancer risk and continual education of health care professionals in this area.

Lastly, this study found *BRCA2* mutation carriers to have a significantly worse survival compared to *BRCA1* carriers. This is an interesting finding but may likely be due to chance since there were no stark differences observed in the tumor subtypes in our study cohort and the power for *BRCA1* carriers was limited. Nevertheless, in a Portuguese cohort of 196 male BC patients, *BRCA2* mutation was found to be associated with poorer overall survival and increased mortality [18]. A recent meta-analysis on *BRCA*-associated prostate cancer risk and mortality also reported an increased mortality in *BRCA2* carriers and concluded that *BRCA2* but not *BRCA1* mutations were associated with higher prostate cancer mortality [15]. Nonetheless, published studies and meta-analyses have reported conflicting results on survival outcomes of patients with *BRCA1/2* mutations [35,36,37], indicating the need for better designed studies to investigate the real effect of the genes on survival.

There are some limitations to the study. The retrospective design means that some data may not have been systematically collected. The sample size is also relatively small. However, due to the rarity of male breast cancer, it takes time to accrue a large sample. As the patients were recruited from the Department of Obstetrics and Gynecology, most patients were from high-risk families, which this could have resulted in selection bias where more *BRCA* mutation carriers and breast cancer cases (and less cases of other cancers) were identified. However, the distribution of gene mutation and cancers identified in this group is rather similar to other reported series of male *BRCA* carriers [5,20]. The study would have also benefited from other comparison groups, i.e., including patients with *BRCA*-variant of uncertain significance or *BRCA*-wild type or matched female *BRCA* mutation carriers for gender differences. 

## 5. Conclusions

Our study has shown that breast and prostate cancers are commonly diagnosed cancers, particularly among *BRCA2* carriers. They also have worse survival and higher frequency of breast cancer diagnosed in the family compared to *BRCA1* carriers. However, not all mutation carriers present with breast cancer or have a family history of cancer to warrant genetic testing. Larger studies are needed to better estimate risks and identify men with *BRCA* mutations, and to further refine existing recommendations regarding early cancer detection in this population.

## Figures and Tables

**Table 1:** Baseline characteristics of men with BRCA1/2-mutations ^1^.

Characteristics	Mutation Type						Total N = 323		P-Value ^2^
	*BRCA1* N = 196 (60.6%)		*BRCA2* N = 120 (37.2%)		*BRCA1* & *BRCA2* N = 7 (2.2%)				
	N	%	N	%	N	%	N	%	
Unaffected	185	94.4	86	71.7	7	100	278	86.1	<0.001
Affected	11	5.6	34	28.3	0	0	45	13.9	<0.001
Type of cancer									
First primary	11	5.6	34	28.3	0	0	45	13.9	<0.001
Second primary	2	1.0	10	8.3	0	0	12	3.7	0.001
Third primary	1	0.5	4	3.3	0	0	5	1.5	0.806
Age at first cancer diagnosis (median year, [range])	61 (58–67)		58 (51–66)		-		-	-	0.283
≤50 years	0	0	5	4.2	-	-	5	0.2	0.200
>50 years	8	4.1	26	21.7	-	-	34	10.5	
Parent of origin ^3^									
Maternal	130	66.3	67	55.8	1	14.3	198	61.3	0.062
Paternal	16	8.2	13	10.8	2	28.6	31	9.6	0.425
Both	0	0	0	0	1	14.3	1	0.3	-
Unknown	50	25.5	40	33.3	3	42.9	93	28.8	-
FDR with breast cancer	88	44.9	73	60.8	7	100	168	52.0	0.008
SDR with breast cancer	66	33.7	71	59.2	7	100	144	44.6	<0.001
FDR or SDR with ovarian cancer	108	55.1	35	29.2	6	85.7	149	46.1	<0.001
Death	6	3.1	12	10.0	1	14.3	19	5.9	0.010

**Abbreviations**: FDR first degree relatives; SDR second degree relatives. ^1^ Column percentages presented. ^2^ Only *BRCA1* and *BRCA2* mutation groups were compared using either Chi-square or Fisher’s test (cell count <5) or Mann-Whitney U-test. ^3^ Only maternal and paternal parent of origin was compared with mutation type.

**Table 2:** Cancer spectrum of BRCA1/2-mutation carriers ^1^.

Type of Cancer	BRCA1 N = 11		BRCA2 N = 34		Total N = 45		P-Value ^2^
	N	%	N	%	N	%	
First primary							
Breast	3	27.3	23	67.6	26	57.8	<0.001
Prostate	3	27.3	4	11.8	7	15.6	0.433
Gastrointestinal	0	0	3	8.8	3	6.7	
Melanoma	2	18.2	1	2.9	3	6.7	
Pancreas	0	0	1	2.9	1	2.2	
Throat	1	9.1	0	0	1	2.2	
Tonsils	1	9.1	0	0	1	2.2	
Lung	1	9.1	1	2.9	2	4.4	
Testis	0	0	1	2.9	1	2.2	
Total	11	100	34	100	45	100	
Second primary							
Breast	0	0	5	50.0	5	41.7	0.007
Prostate	1	50.0	2	20.0	3	25.0	0.560
Melanoma	0	0	1	10.0	1	8.3	
Liver	1	50.0	0	0	1	8.3	
Pancreas	0	0	1	10.0	1	8.3	
Chronic lymphatic leukemia	0	0	1	10.0	1	8.3	
Total	2	100	10	100	12	100	
Third primary							
Breast	0	0	2	50.0	2	40.0	
Prostate	0	0	1	25.0	1	20.0	
Gastrointestinal	0	0	1	25.0	1	20.0	
Liver	1	100	0	0	1	20.0	
Total	1	100	4	100	5	100	

^1^ Column percentages presented. ^2^ Fisher’s test.

**Table 3:** Primary breast tumor characteristics by mutation type ^1^.

Characteristics	BRCA1 (N = 3)		BRCA2 (N = 26)	
Laterality	N	%	N	%
Unilateral	3	100	21	80.8
Bilateral	0	0	0	0
Unknown	0	0	5	19.2
**Side affected**				
Left	3	100	13	50.0
Right	0	0	8	30.8
Unknown	0	0	5	19.2
**Behaviour**				
Invasive	1	33.3	15	57.7
In-Situ	0	0	0	0
Unknown	2	66.7	11	42.3
**Histological Subtype**				
Ductal	1	33.3	12	46.2
Lobular	0	0	1	3.8
Medullary	0	0	0	0
Other ^2^	0	0	2	7.7
Unknown	2	66.7	11	42.3
**Grade**				
G1	0	0	2	7.7
G2	0	0	8	30.7
G3	1	33.3	6	23.1
Unknown	2	66.7	10	38.5
**Tumor Size**				
T1	1	33.3	9	34.6
T2	0	0	7	26.9
T3	0	0	0	0
Unknown	2	66.7	10	38.5
**Node Status**				
N0	1	33.3	10	38.5
N1	0	0	4	15.4
N2	0	0	1	3.8
N3	0	0	1	3.8
Unknown	2	66.7	10	38.5
**Metastases**				
M0	0	0	5	19.2
M1	0	0	4	15.4
Unknown	3	100	17	65.4
**Hormone Receptor Status**				
ER ^3^				
Positive	1	33.3	14	53.8
Negative	0	0	1	33.3
Unknown	2	66.7	11	42.3
PR ^3^				
Positive	1	33.3	13	50.0
Negative	0	0	3	11.5
Unknown	2	66.7	10	38.5
HER2-Status				
Positive	0	0	4	15.4
Negative	1	33.3	11	42.3
Unknown	2	66.7	11	42.3
Ki-67				
>20%	0	0	8	30.8
<20%	0	0	5	19.2
Unknown	3	100	13	50.0
**Type of Surgery**				
Unilateral mastectomy	1	33.3	16	61.5
Unknown	2	66.7	10	38.5
**Prophylactic Surgery**				
Prophylactic contralateral mastectomy	0	0	3	11.5
Unknown	3	100	23	88.5

**Abbreviations**: ER estrogen receptor, PR progesterone receptor, HER2 human epidermal growth factor receptor 2. ^1^ Column percentages presented. ^2^ One papillary and one tubular-ductal carcinoma. ^3^ Estrogen and progesterone receptor status are reported as negative/positive or as intensity score consisting of negative/weak/moderate/strong. Weak/moderate are recategorized as negative and strong are recategorized as positive.

**Table 4:** Age at first cancer diagnosis according to cancer site and mutation status.

First Cancer Diagnosis	Mutation Type ^1^				Total N = 316		*p*-Value ^2^
	*BRCA1* N = 196		*BRCA2* N = 120				
	N (%)	Age at Diagnosis (Median, [Range])	N (%)	Age at Diagnosis (Median, [Range])	N (%)	Age at Diagnosis (Median, [Range])	
Breast	3 (1.5)	62 (59–81)	23 (19)	57 (39–82)	26 (8.2)	58 (39–82)	0.170
Prostate	2 (1.0)	58 (56–60)	3 (2.5)	52 (50–63)	5 (1.6)	56 (50–63)	0.619
Others ^3^	3 (1.5)	67 (58–68)	5 (4.2)	65 (40–88)	8 (2.5)	66 (40–88)	0.255

^1^ Column percentages presented. ^2^ Fisher’s test. ^3^ Other cancers include gastrointestinal, melanoma, pancreas, throat, tonsils, lung and testis.

**Table 5:** Family history of cancer and parent of origin by first primary cancer and mutation type ^1^.

Characteristics	BRCA1 N = 196		BRCA2 N = 120		Total N = 316		*p*-Value ^2^
**Family History of Cancer**	N	%	N	%	N	%	
Breast cancer							
Yes	1	33.3	14	60.9	15	57.7	0.556
No	2	66.7	9	39.1	11	42.3	
Other cancers							
Yes	4	50.0	8	72.7	12	63.2	0.377
No	4	50.0	3	27.3	7	36.8	
No cancer							
Yes	83	44.9	51	59.3	134	49.4	0.027
No	102	55.1	35	40.7	137	50.6	
**Parent of origin**							
Breast cancer							
Maternal	2	66.7	9	39.1	11	42.3	0.670
Paternal	0	0	2	8.7	2	7.7	
Unknown	1	33.3	12	52.2	13	50.0	
Other cancers							
Maternal	3	37.5	5	45.5	8	42.1	0.689
Paternal	2	25.0	1	9.1	3	15.8	
Unknown	3	37.5	5	45.5	8	42.1	
No cancer							
Maternal	125	67.6	53	61.6	178	65.7	0.475
Paternal	14	7.6	10	11.6	24	8.9	
Unknown	46	24.9	23	26.7	69	25.5	

^1^ Column percentages presented. ^2^ Chi-square or Fisher’s test.

## Data Availability

The data presented in this study are available upon request from the corresponding author. The data are not publicly available due to privacy and ethical reasons.

## Supplementary Materials

The following are available online at https://www.mdpi.com/article/10.3390/jpm11090917/s1, Table S1: Characteristics of patients with two breast cancer diagnoses.
