# Supplementary material for: Cancer Spectrum, Family History of Cancer and Overall Survival in Men with Germline BRCA1 or BRCA2 Mutations
Source: J Pers Med. 2021 Sep 15;11(9):917. doi: 10.3390/jpm11090917 (PMC8466243; doi:10.3390/jpm11090917)
Supplement: Supplementary file 1 [file jpm-11-00917-s001.zip › jpm-1351889-supplementary.pdf]

Journal of Personalized Medicine

MDPI

Supplementary Table S1. Characteristics of patients with two breast cancer diagnoses.

	**Patient 1**	**Patient 2**	**Patient 3**	**Patient 4**
Mutation	BRCA2	BRCA2	BRCA2	BRCA2
Mutation-Type	Frameshift	Splice Site	Missense	Frameshift
Mutation-Location	c.3847_3848del	c.7617+2T>G	c.9371A>T	c.5544delT
Family history				
FDR with BC	1	0	2	0
SDR with BC	0	0	0	0
Year of diagnosis	1996	1974	2011	2008
Age at diagnosis				
First BC	60	42	68	59
Second BC	65	77	76	61
Laterality				
First BC	unilateral	unilateral	unilateral	unilateral
Second BC	unilateral	unilateral	unilateral	n.a
Side affected				
First BC	left	left	left	right
Second BC	right	right	right	n.a.
Behavior				
First BC	n.a.	n.a.	invasive	invasive
Second BC	invasive	invasive	invasive	n.a.
Morphology				
First BC	n.a.	n.a.	ductal	ductal
Second BC	ductal	ductal	ductal	n.a.
Grade				
First BC	n.a.	n.a.	G2	G3
Second BC	G2	G3	G3	n.a.
Tumor size				
First BC	T2	n.a.	T2	n.a.
Second BC	T1	T2	T2	n.a.
Node status				
First BC	N1	n.a.	N0	n.a.
Second BC	N0	N3	N0	n.a.
Metastases				
First BC	M0	M0	M0	M1 (Brain)
Second BC	M1 (Bone)	M1 (Liver)	M0	n.a.
ER status				
First BC	n.a.	n.a.	positive	positive
Second BC	positive	positive	positive	n.a.
PR status				
First BC	n.a.	n.a.	positive	negative
Second BC	positive	positive	positive	n.a.
HER2 status				
First BC	n.a.	n.a.	positive	n.a.
Second BC	n.a.	positive	negative	n.a.
Ki-67				
First BC	n.a.	n.a.	n.a.	n.a.
Second BC	n.a.	40	n.a.	n.a.

Type of surgery				
First BC	Modified radical mastectomy	Modified radical mastectomy	Modified radical mastectomy	Modified radical mastectomy
Second BC	Modified radical mastectomy	Modified radical mastectomy	Modified radical mastectomy	n.a.
Age at Death	76	81	Alive	Alive
Cause of Death	Generalized metastatic spread - **Breast Cancer C50.9**	Cardiac arrest due to **Breast Cancer C50.9**	-	-

Abbreviations FDR first-degree relative(s); SDR second-degree relative(s); BC breast cancer; n.a. not available; ER estrogen receptor; PR progesterone receptor; HER2 receptor human epidermal growth factor receptor2.
